# Lipid Profiles of Human Serum Fractions Enhanced with CD9 Antibody-Immobilized Magnetic Beads

**DOI:** 10.3390/metabo12030230

**Published:** 2022-03-05

**Authors:** Suzumi M. Tokuoka, Yoshihiro Kita, Masaya Sato, Takao Shimizu, Yutaka Yatomi, Yoshiya Oda

**Affiliations:** 1 Department of Lipidomics, Graduate School of Medicine, The University of Tokyo, Hongo 7-3-1, Bunkyo-ku, Tokyo 113-8654, Japan; stokuoka@m.u-tokyo.ac.jp (S.M.T.); kita@m.u-tokyo.ac.jp (Y.K.); tshimizu@ri.ncgm.go.jp (T.S.); 2 Department of Clinical Laboratory Medicine, Graduate School of Medicine, The University of Tokyo, Hongo 7-3-1, Bunkyo-ku, Tokyo 113-8654, Japan; masayasato0407@gmail.com (M.S.); yatomiy-lab@h.u-tokyo.ac.jp (Y.Y.); 3 National Center for Global Health and Medicine, Department of Lipid Signaling, Toyama 1-21-1, Shinjuku-ku, Tokyo 162-8655, Japan

**Keywords:** immunoprecipitation, CD9, lipidomics, human serum

## Abstract

Blood samples are minimally invasive and can be collected repeatedly, but they are far from the site of disease and the target molecules are diluted by the large amount of blood. Therefore, we performed lipidomics using immunoprecipitation as a method to enrich specific fractions of serum. In this study, a CD9 antibody was immobilized on magnetic beads to enrich CD9-containing components in the serum for lipidomics. The percentages of phospholipids recovered from serum by methanol and isopropanol extractions were not significantly different, but triglycerides were barely recovered from serum by methanol extraction, requiring the use of isopropanol. However, once the serum was enriched with CD9 magnetic beads, triglycerides, and phospholipids were recovered at similar levels in both methanol and isopropanol extractions. Therefore, it is possible that the triglyceride fraction of the whole serum and the triglyceride fraction were enriched in CD9 magnetic beads differ in localization and properties. In addition, the variation per disease was small in general serum lipidomics; however, the difference per disease appeared larger when CD9 magnetic bead enrichment was employed.

## 1. Introduction

Lipidomics is a field of research that focuses on whole lipids in cells, often using high-performance liquid chromatography (HPLC) or gas chromatography as a separation method and mass spectrometry (MS) as a detection method. There are thousands of lipids in the human body, and the expression and localization of lipid molecules can change in response to changes in the cellular environment due to factors such as exercise, diet, and health conditions. Measuring these changes and understanding lipid-related pathways are important for understanding cellular metabolism, and lipids are also important biomarkers when considering disease mechanisms [1,2,3]. In the context of studies on human diseases, directly analyzing human samples is simpler than investigating cells or animals. While cancer is relatively easy to investigate, as we can obtain samples from diseased tissues, there are many diseases, such as psychiatric disorders, where the pathological sites are unclear or difficult to obtain. In addition, biopsies and other biological samples that are difficult to collect repeatedly are unsuitable for studying changes over time. 

Blood samples, such as serum and plasma, are taken by minimally invasive means, are repeatable, and reflect the circulation of the body; therefore, they are thought to reflect the states of various diseases to a certain extent [4]. Therefore, blood is often used for biomarker discovery and various lipid markers have been investigated [5,6,7,8]. However, one of the disadvantages of blood samples is that they are often far from the disease site, and the large volume of blood dilutes the molecules originating from the pathological tissue. Therefore, the density gradient centrifugation methods [9,10] and the immunoprecipitation methods [11,12,13] are known to concentrate only specific fractions in serum or plasma. Since the ultracentrifugation method is not suitable for the measurement of multiple samples, we decided to explore the possibility of the immunoprecipitation method. The fractionation of serum components by immunoprecipitation has recently been used in proteomics [11,12,13], but it has rarely been used in lipidomics.

The tetraspanin superfamily is a highly conserved protein family, with at least 33 members identified in humans, including CD9, CD63, and CD81 [14]. In particular, CD9 is highly expressed on major subsets of leukocytes (e.g., B and T cells, monocytes, and macrophages), platelets [15,16,17] and endothelial cells [18]. In many diseases, CD9 is considered functionally important [19]. 

We immobilized CD9 antibodies on magnetic beads and examined the lipid composition of serum enriched by CD9-magnetic beads to determine which lipid classes are more likely to be enriched by these CD9-magnetic beads, and how the results differ from general serum lipidomics.

## 2. Results

**Figure 1:**
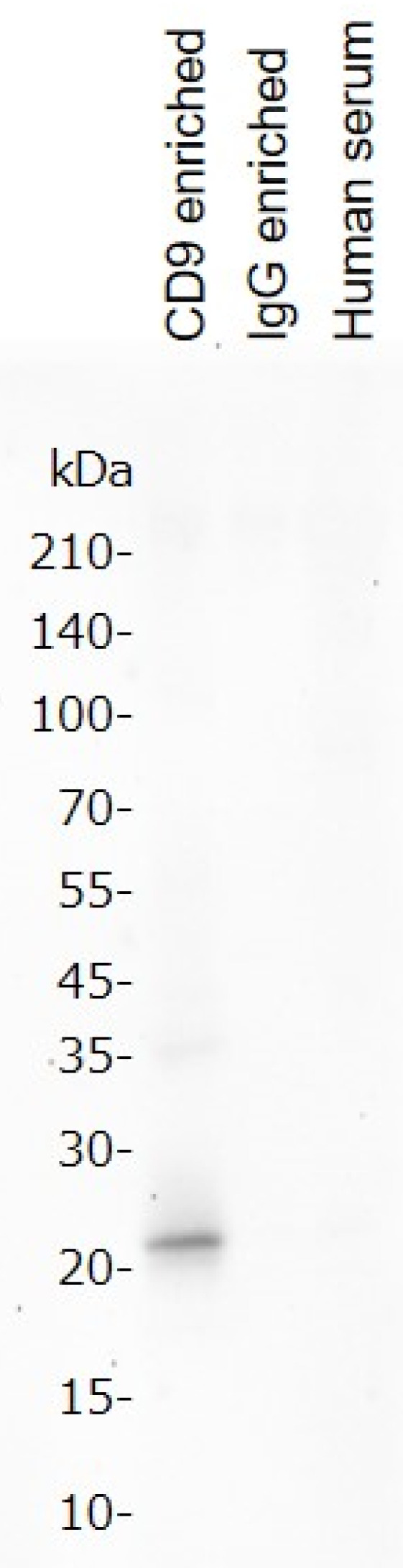
Western blot for CD9 enrichment in human serum.

We tested whether CD9 antibody-immobilized magnetic beads could be used to enrich CD9 from human serum. Non-specific immunoglobulin G (IgG)-immobilized magnetic beads were used for comparison. One hundred microliters of human serum diluted threefold in phosphate buffered saline (PBS) was mixed with a CD9 antibody or IgG immobilized magnetic beads slowly for 2 h at 4 °C. The beads were collected using a magnet, the supernatant was discarded, and the beads were washed with 500 µL PBS. Binding components were eluted with sodium dodecyl sulfate (SDS)-polyacrylamide gel electrophoresis (PAGE) sample buffer, separated by SDS-PAGE, and subjected to western blotting using a CD9 antibody. The results are shown in Figure 1. As expected, CD9 antibody-immobilized magnetic beads were able to enrich CD9 from human serum, whereas IgG-immobilized magnetic beads could not detect CD9.

The molecular weights are shown on the left. From left to right, the lanes are for the CD9 antibody-enriched, IgG-enriched, and human serum fractions (without enrichment). The samples were separated by gel electrophoresis, the bands were stained with the CD9 antibody, and detected by chemiluminescence.

**Figure 2:**
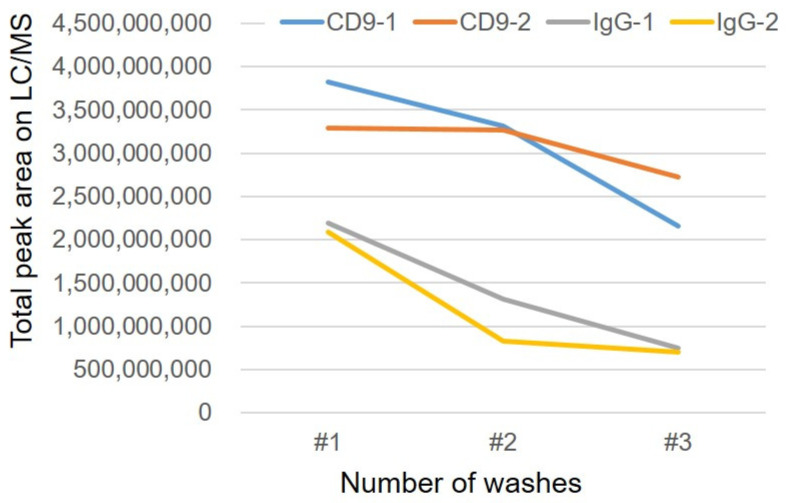
Number of washes of magnetic beads and lipid adsorption. After mixing the magnetic beads with human serum, the number of times the beads were washed with PBS is on the horizontal axis and the total area of the lipid peak detected by LC/MS is shown on the vertical axis. PBS is used for washing and MeOH is used for elution. The CD9 antibody-immobilized magnetic beads and IgG-immobilized magnetic beads were tested twice, and the results of each experiment are shown in the graphs.

**Figure 3:**
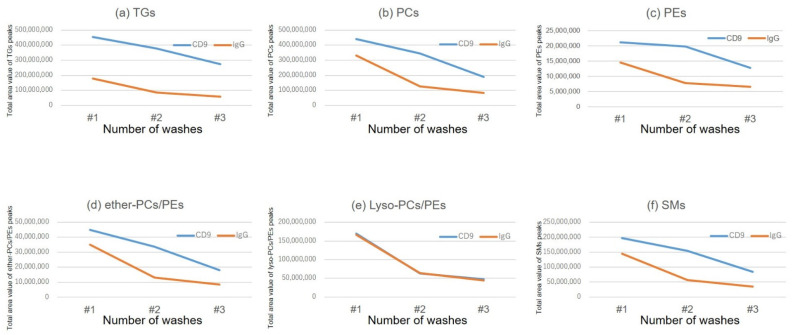
Number of washes of magnetic beads and adsorbed amount of lipids by class. (**a**) Tgs, (**b**) PCs, (**c**) PEs, (**d**) ether PCs and ether Pes, (**e**) lysoPCs ans lysoPEs, (**f**) SMs.

The difference in the amount of lipids enriched by CD9 magnetic beads and IgG magnetic beads was larger when washed twice with PBS (Figure 2). Figure 3 shows the results of lipid enrichment according to lipid class.

**Figure 4:**
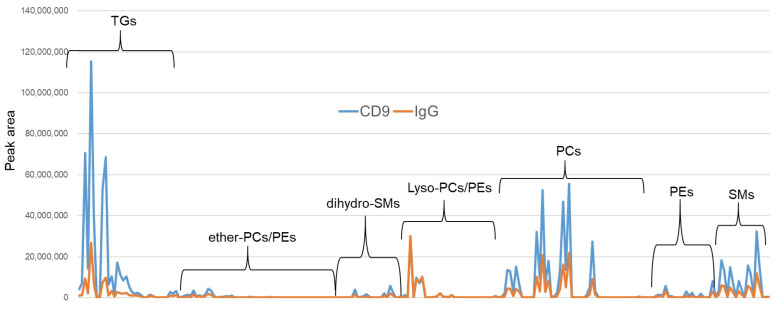
Profiles of lipid components in human serum enriched with magnetic beads. Lipids are classified by class and arranged on the horizontal axis, and the vertical axis shows the peak area of each lipid molecule. Blue shows the lipid profile enriched by CD9 antibody-immobilized magnetic beads and orange shows the lipid profile enriched by IgG-immobilized magnetic beads. TG, triglyceride; PC, phosphatidylcholine; PE, phosphatidylethanolamine; SM, sphingomyelin.

After mixing the magnetic beads with human serum, the number of times the beads were washed with PBS (horizontal axis) and the total peak area detected by LC/MS by lipid class is shown on the vertical axis. The CD9 antibody-immobilized magnetic beads and IgG-immobilized magnetic beads were operated twice, and the average values are shown in the graph. (LC/MS, liquid chromatography/mass spectrometry; PBS, phosphate buffered saline; PC: phosphatidylcholine, PE: phosphatidylethanolamine, SM: sphingomyelin; TG, triglyceride.) In Figure 4, the lipid profiles obtained after two PBS washes are shown. For triglycerides (TGs), the difference between CD9 antibody- and IgG-conjugated magnetic beads was larger than that for other lipid classes, whereas for lysophospholipids, IgG-conjugated magnetic beads adsorbed almost as much as CD9-conjugated ones. In this study, it was found that lipids were adsorbed on the plastic tubes used in addition to the magnetic beads, and the lipids adsorbed on the containers were also released by adding an organic solvent when eluting the lipids from the magnetic beads. Therefore, the magnetic beads were transferred to a new plastic tube during the last washing step before elution.

**Figure 5:**
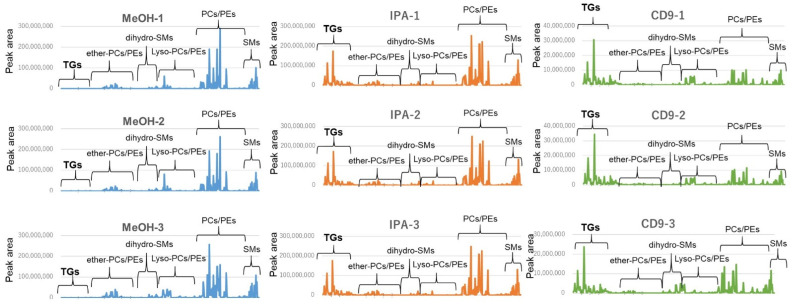
Comparison of lipid profiles in different organic solvents used for lipid extraction from human serum.

The Bligh and Dyer or Folch procedure for the extraction of lipids is the gold standard, but it is not easy to operate because it is a two-layer separation and lipids are in the lower layer. Protein removal by addition of organic solvents is simple, but the recovery rate of lipids varies greatly depending on the solvent [20,21]. To confirm this, the lipid profiles of serum extracted with 10 times the volume of methanol (MeOH) and isopropanol (IPA) are shown in Figure 5 (left and middle columns, repeated 3 times). The extraction efficiency of most phospholipids did not differ substantially between MeOH and IPA; however, IPA extraction of TG resulted in each peak area about 100 times larger than that of MeOH extraction. When serum was enriched with CD9 magnetic beads, TGs were extracted from human serum as well as other phospholipids (Figure 5 (right columns, repeated 3 times)). 

Lipids are grouped by each lipid class and arranged on the horizontal axis, and the vertical axis shows the peak area of each lipid molecule. The left column (blue) shows the lipid profile of direct MeOH extraction of serum, the middle column (orange) shows the lipid profile of direct IPA extraction of serum, and the right column (green) shows the lipid profile of CD9 antibody-immobilized magnetic bead extraction of serum. Each extraction method was repeated three times, and their lipid profiles are shown in vertical order (from top to bottom: −1, −2, −3). (TG: Triglyceride, PC: Phosphatidylcholine, PE: Phosphatidylethanolamine, SM: Sphingomyelin, MeOH: Methanol, IPA: Isopropanol).

This result suggests that the CD9 magnetic beads may enrich low-density lipoprotein (LDL), which is present in the human serum, together with TGs and phospholipids. Therefore, we checked whether LDL and HDL were enriched by the CD9 magnetic beads. The cholesterol contents of HDL and LDL in the serum were 1.8 mM and 2.7 mM, respectively. However, in CD9- and IgG-immobilized magnetic bead enriched fractions, both LDL and HDL were below the detection limit (<50 µM), which was less than one tenth of that in serum.

We investigated the possibility that the lipid profile enriched by CD9-immobilized magnetic beads might differ depending on the disease. We collected samples from 18 patients each diagnosed with Alzheimer’s disease, Parkinson’s disease, cerebral stroke, major depression, or schizophrenia who visited the University of Tokyo Hospital for examination, and prepared three mixed samples for each disease by mixing six serum samples for each disease (Table 1). 

**Figure 6:**
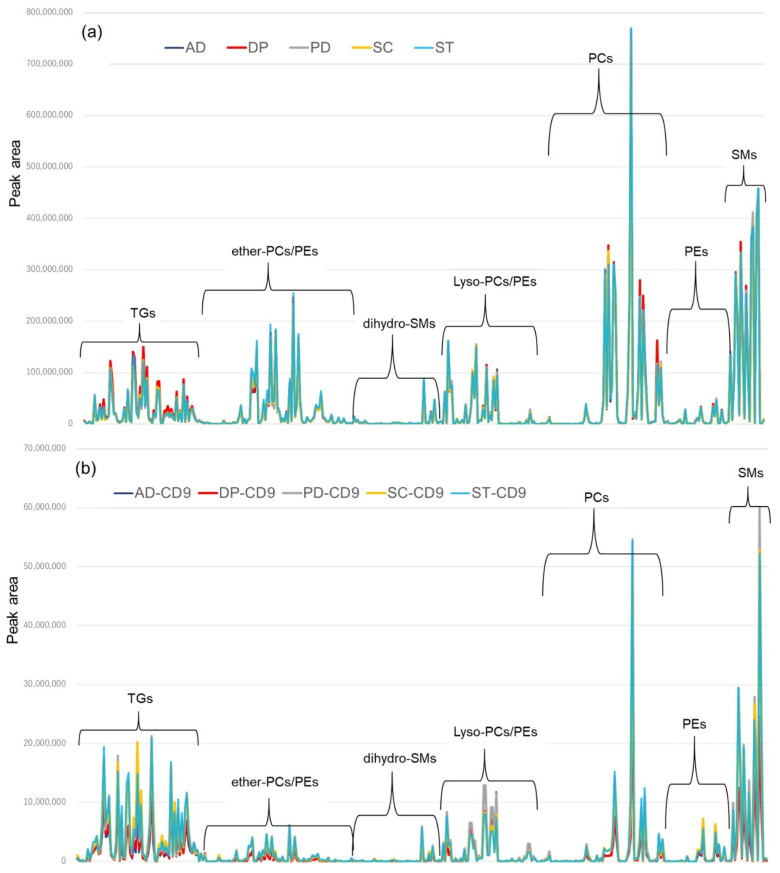
Serum lipid profile by disease. Lipids are classified by class and arranged on the horizontal axis, and the vertical axis shows the peak area of each lipid molecule. Dark blue indicates Alzheimer’s disease (AD), red indicates major depression (DP), gray indicates Parkinson’s disease (PD), yellow indicates schizophrenia (SC), and light blue indicates cerebral stroke (ST). All values are the average of the three groups. (**a**) Lipid extraction directly from human serum, (**b**) Lipid extraction after enrichment of human serum with CD9 antibody-immobilized magnetic beads. TG: triglyceride, PC: phosphatidylcholine, PE: phosphatidylethanolamine, SM: sphingomyelin.

The results of IPA extraction of serum and lipid profile for each serum sample (graphical representation of each peak area value [average value for each disease]) and the lipid profile enriched on CD9 magnetic beads are shown in Figure 6. Serum lipidomics showed similar profiles regardless of the type of disease (Figure 6a); however, enrichment with CD9 magnetic beads may have resulted in different profiles for different diseases (Figure 6b). The bar graph with error bars (by disease) is shown in Supplementary Materials Figure S1.

**Figure 7:**
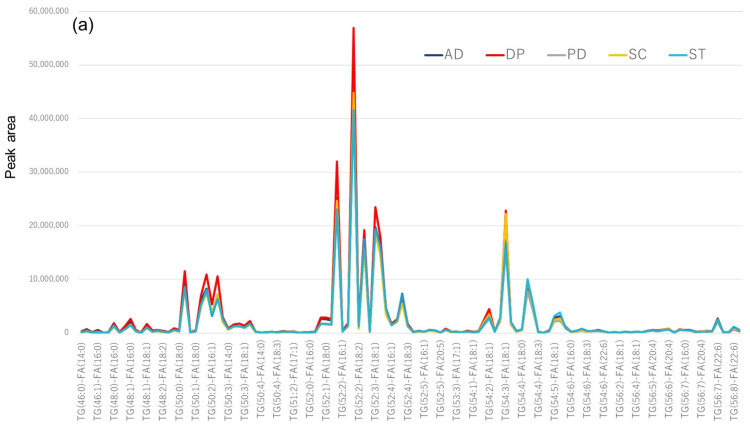

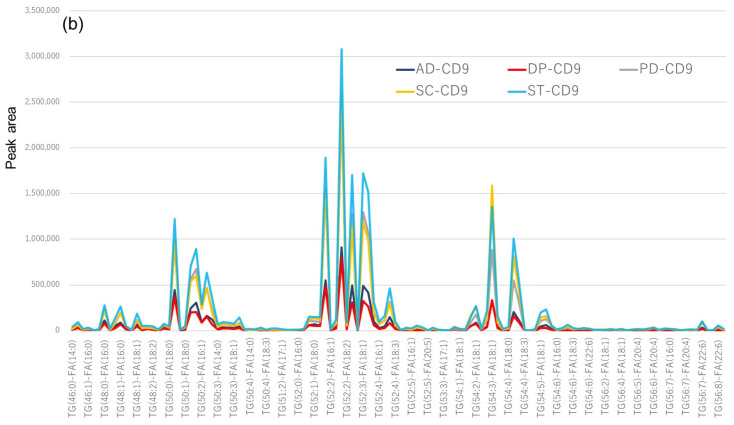
Serum triglyceride profile by disease. Lipids are classified by class and arranged on the horizontal axis, and the vertical axis shows the peak area of each triglyceride molecule. Dark blue indicates Alzheimer’s disease (AD), red indicates major depression (DP), gray indicates Parkinson’s disease (PD), yellow indicates schizophrenia (SC), and light blue indicates cerebral stroke (ST). TG (W:X)-FA (Y:Z) represents the components containing fatty acid Y:Z (carbon number Y, unsaturated bond number Z) among triglycerides whose total sum of three carbons in the fat chain is W and the total sum of unsaturated bonds constituting the fat chain is X. All profiles are the average of the three groups. (**a**) Triglycerides were extracted by isopropanol directly from human serum, (**b**) Triglycerides were extracted by CD9 antibody-immobilized magnetic beads from human serum.

Because enrichment by CD9 magnetic beads is thought to have a significant effect on TG (Figure 3, Figure 4 and Figure 5), we focused our measurements on TG, and the results are shown in Figure 7. In the serum without CD9 magnetic beads (IPA extraction), there is a little more TG in major depression (red line), but there is no significant difference among the five diseases (Figure 7a). However, in the TG profile of serum enriched with CD9 magnetic beads, there appears to be a large difference among the five diseases. Bar graphs with error bars (by disease) are shown in Supplementary Materials Figure S2.

**Figure 8:**
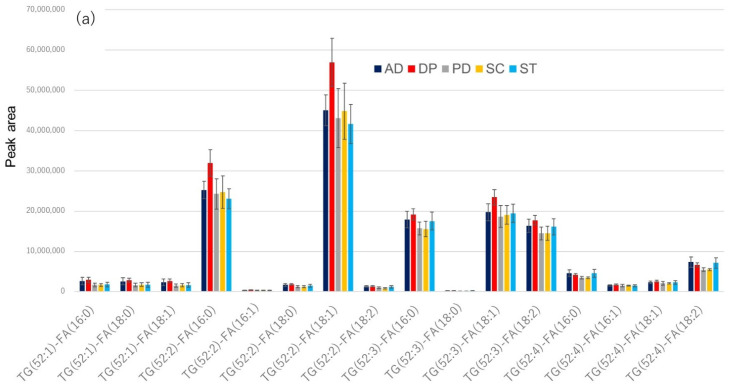

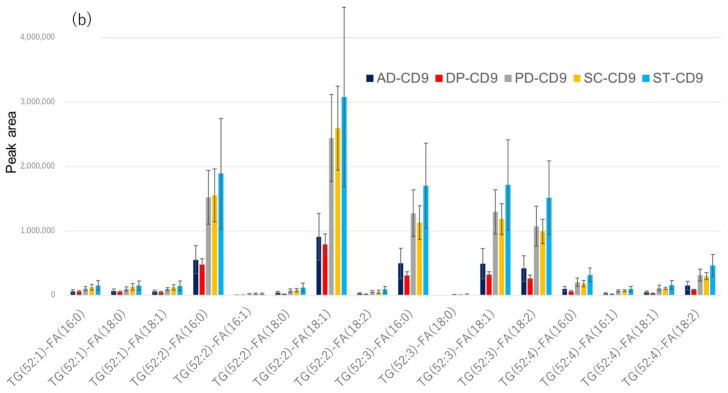
Bar graph of serum triglyceride profile by disease, enlarging the horizontal axis around TG(52:1) to TG(52:4) in Figure 7. The colors and abbreviations are the same as in Figure 7. (**a**) TG profiles based on IPA extraction directly from serum, (**b**) TG profiles based on serum samples enriched by CD9 magnetic beads.

The bar graphs from TG(52:1) to TG(52:4) in Figure 7 were expanded by disease (Figure 8). In the case of IPA extraction of serum, the TG profiles did not appear to differ much among the five diseases (Figure 8a). However, in the TG profiles of serum enriched by CD9 magnetic beads, Alzheimer’s disease and major depression seem to be less abundant than the other three diseases (Figure 8b). Figure S3 shows an enlarged bar graph of PC(30:0) to PC(40:9), where Alzheimer’s disease and major depression appear to be different from the other three diseases in the PC profiles of sera enriched with CD9 magnetic beads as well as TG. In the case of LPC, however, the profiles did not differ much among the five diseases, whether the serum was extracted by IPA or enriched with CD9 magnetic beads (Figure S4). This may be because no difference in specificity was observed between CD9 and IgG in the case of LPC (Figure 3e and Figure 4). When the results in Figure 8b were evaluated by ANOVA (Post-hoc analysis: Tukey) for 5-group comparison, no molecules had an Adjusted p-value (FDR) less than 0.05. The number of measurements may have been too small and the setting of the five diseases to be compared may have been unreasonable.

## 3. Discussion

Serum and plasma are relatively easy samples to use for routine diagnosis, and since they circulate throughout the body, they are thought to reflect various disease states [4]; at the same time, the markers from lesion sites are diluted by a large amount of blood. Therefore, methods to concentrate specific fractions of serum or plasma by immunoprecipitation or other methods have been tried [11,12,13]. CD9, a member of the tetraspanin superfamily, has a variety of biological activities and is considered to be functionally crucial [19]. CD9 is known to accumulate in EVs [22,23]. In this study, we focused on CD9 and investigated the components enriched by magnetic beads with an immobilized CD9 antibody. We found that TGs were easily enriched by the CD9 magnetic beads. LDL is co-purified when EVs are isolated [24,25,26]. Therefore, we examined the possibility that LDL was enriched by CD9 magnetic beads and confirmed that was not the case. CD9 is also highly expressed in platelets [15,16,17]; however, since we used serum in this study, we believe that platelet contamination would be minimal. Regardless, we could not completely rule out the presence of platelet fragments and LDL. We observed that TGs in the serum were not easily recoverable by MeOH extraction, but when CD9 magnetic beads were used, TGs and various phospholipids could be easily measured after MeOH extraction. In the TG profile, the pattern obtained by IPA extraction from serum was different from that obtained by CD9 magnetic bead enrichment, suggesting that TGs enriched by CD9 magnetic beads may be present in a different fraction from MeOH-insoluble and IPA-soluble TGs in the serum. When we compared the TG profiles of five different diseases, we found that there were almost no differences in the amounts of TGs extracted from serum by IPA among the examined samples; however, the amount of TGs enriched by CD9 magnetic beads may vary depending on the disease. In the present study, considering the cost of antibody-immobilized magnetic beads and the complexity of the operation, the samples of 18 patients with each disease were mixed into three pooled samples of 6 patients each, but to verify the differences according to disease, it is necessary to improve cost and throughput. In addition, performing small-molecule omics using mass spectrometry, such as lipidomics and metabolomics, is often not sufficiently reproducible, and there are concerns about peak identification and purity [24,25,26,27]. Therefore, instead of discussing each peak or lipid molecule, we examined pattern changes in each lipid class. Even if there is substantial uncertainty for individual molecules, such ambiguous results are highly likely to occur randomly and would be more credible if they varied in a population, such as a specific pathway or network [28,29,30,31]. This study is positioned as an exploratory screening, and after finding candidate target molecules, we will prepare stable isotope-labeled internal standards, perform analytical validation, and then proceed to the validation process of CD9 antibody magnetic bead enrichment fractions. When serum was extracted by IPA, neither TGs nor phospholipid profiles seemed to differ significantly among the five diseases, but when serum was enriched with CD9 antibody magnetic beads, TGs (Figure 8) and PCs (Figure S3) in Alzheimer’s disease and major depression seemed to be lower than those in the other three diseases. However, no such trend was observed for LPCs (Figure S4). Since lysophospholipids are not specific for CD9 antibodies and are enriched to the same extent in IgG magnetic beads, non-specific binding may have a significant effect on LPCs. Usually, it is difficult to completely avoid non-specific adsorption in lipid analysis. It was confirmed that the inter-disease profiles of TG and PC, which differed by serum enrichment with CD9 antibody magnetic beads and IgG magnetic beads, were both similar, but the inter-disease profile of LPC, which did not differ by CD9/IgG magnetic beads enrichment (Figure 3e and Figure 4), was different from that of TG and PC. This may have been due to non-specific adsorption. It should be emphasized that the results of this study showed that CD9 antibody magnetic beads can easily concentrate TGs in serum. Here, we examined the lipid components in serum enriched with CD9 antibody-immobilized magnetic beads and found that TGs, which are not easily extracted by MeOH, were enriched (CD9-enriched TGs) and showed a different pattern from TGs in serum components (pan-TGs). Whether this difference between CD9-enriched TGs and pan-TGs is a biologically meaningful fraction needs to be investigated in the future. Although this is an exploratory study, we plan to select appropriate diseases, find candidate target molecules, and confirm the certainty of the results in the future.

## 4. Materials and Methods

### 4.1. Reagents

The CD9 antibody (clone number 12A12) was obtained from Cosmo Bio Co. (Tokyo, Japan). HDL and LDL/VLDL cholesterol assay kits were purchased from Cell Biolabs, Inc. (San Diego, CA, USA). HEPES, MES, ethanolamine, EDTA·2Na, polyoxyethylene lauryl ether, and normal mouse IgG were obtained from Sigma-Aldrich (Saint Louis, MO, USA). Lipid standards were obtained from the Cayman Chemical Company (Ann Arbor, MI, USA) and Avanti (Birmingham, AL, USA). Special grade reagents of ammonium bicarbonate and PBS and HPLC grade solvents of MeOH, IPA, and acetonitrile were purchased from Fujifilm (Osaka, Japan), and ultrapure water was obtained from a Milli-Q System (Burlington, MS, USA). 

### 4.2. Serum Samples

This study was conducted with the approval of the ethics committee of the University of Tokyo Hospital and in compliance with the relevant guidelines and regulations. A pooled serum sample was generated by mixing 300 randomly selected patient residual samples after completion of the requested clinical laboratory tests at the University of Tokyo Hospital (Institutional Research Ethics Committee of the Faculty of Medicine, University of Tokyo approval number: 2019022NI). Japanese neurodegenerative and psychiatric disease specimens were obtained from residual samples from patients who had been examined at the University of Tokyo Hospital (Institutional Research Ethics Committee of the Faculty of Medicine, The University of Tokyo approval number: 2019068NI). Informed consent for participation in the study was obtained using an opt-out process on the web page of the University of Tokyo Hospital. The investigations were carried out in accordance with the rules of the Declaration of Helsinki of 1975 (https://www.wma.net/what-we-do/medical-ethics/declaration-of-helsinki/, our last access was 24 November 2021), revised in 2013. Diagnoses in the electronic medical record system were recorded within two years before the date of blood sampling was used. All samples were stored at −80 °C prior to analysis. 

### 4.3. Preparation of CD9 Antibody- and IgG-Immobilized Magnetic Beads

First, 100 µL (2 mg) of HM NHS beads were added to a 1.5 mL Eppendorf tube, which was centrifuged at 10,000× *g* for 5 min, before the supernatant was discarded. Next, 200 µL of MeOH was added, the mixture was suspended well, it was centrifuged at 10,000× *g* for 5 min, and the supernatant was discarded. Next, 200 µL of 25 mM MES buffer (pH 6) was added to disperse the beads, which were centrifuged at 10,000× *g* for 5 min before the supernatant was discarded. We then added 100 µL each of 1 mg/mL CD9 antibody and IgG to Eppendorf tubes containing the magnetic beads. We added 100 µL of 25 mM MES buffer (pH 6) and suspended the mixture. The mixture was agitated slowly at 4 °C for 4 h. The mixture was then centrifuged at 10,000× *g* for 5 min, and the supernatant was discarded. Subsequently, 300 µL of a solution with final concentrations of 0.1 M HEPES buffer, 1 M ethanolamine, and 1% polyoxyethylene was adjusted to pH 8 with hydrochloric acid and added to the tube containing the magnetic beads, which was mixed slowly at 4 °C overnight. The sample was centrifuged at 10,000× *g* for 5 min, the supernatant was discarded and 300 µL of 25 mM HEPES solution (pH 8) containing 2 mM EDTA was added. The magnetic beads were washed, centrifuged at 10,000× *g* for 5 min, and the supernatant was discarded. Finally, we added 400 µL of 25 mM HEPES solution (pH 8) containing 2 mM EDTA and stored the tube at 4 °C. The protein immobilized bead concentration was approximately 0.1 mg/20 µL.

### 4.4. Serum Enrichment with CD9 Antibody and IgG-Immobilized Magnetic Beads

Then, 200 µL of PBS was added to 100 µL of serum, and 7 µL of protein-immobilized magnetic beads were added while suspending them well. The mixture was slowly mixed for 2 h at 4 °C. The water adhering to the back cover of the tube was also dropped into the tube, the magnetic beads were collected using a magnet, and the supernatant was discarded. After adding 500 µL of PBS, the magnetic beads were mixed well, collected with a magnet, and discarded. Another 500 µL of PBS was added and the magnetic beads were mixed well. After transfer to a new tube, the magnetic beads were collected using a magnet, and the supernatant was discarded. MeOH or IPA (50 µL) was added and mixed, the magnetic beads were collected with a magnet, and the supernatant was analyzed by LC/MS.

### 4.5. Liquid Chromatography/Mass Spectrometry Measurements

The LC and MS measurement conditions were as follows: Serum samples were subjected to a Nexera UHPLC system and LCMS-8060 triple quadrupole mass spectrometer (Shimadzu Co., Kyoto, Japan). An Acquity UPLC BEH C8 column (1.7 µm, 2.1 × 100 mm; Waters) was used with the following mobile phase compositions: 5 mM NH_4_HCO_3_/water (mobile phase A), acetonitrile (mobile phase B), and isopropanol (mobile phase C). The pump gradient was programmed as follows [time (%A/%B/%C)]: 0 min (95/5/0), to 8 min (70/30/0), 16 min (30/35/35), 28 min (6/47/47), 35 min (6/47/47), 35.1 min (95/5/0); it was then held for 38 min for equilibration. The flow rate was 0.35 mL/min, and the column temperature was 47 °C. The injection volume was 5 µL. SRM analysis was performed using positive/negative ion-switching mode ESI, with a collision energy of 46 eV. All data were analyzed using Microsoft Excel 2016. 

### 4.6. Western Blot

For serum-enriched fractions using the CD9 antibody and IgG-immobilized magnetic beads, 30 µL of SDS-PAGE sample buffer was added after washing with PBS. Serum was diluted 50-fold with SDS-PAGE sample buffer because the of the excessive amount of protein. They were heated at 95 °C for 3 min. Fifteen microliters per lane was added to the gel and separated by SDS-PAGE (10–20% gradient gel), and then transferred to a polyvinylidene difluoridemembrane (pore size 0.2 µm). After blocking and washing with 1% skim milk, biotin-labeled CD9 antibody (1 µg/mL) was used as the primary antibody and Neutralite Avidin-HRP (Horseradish peroxidase) (Southern Biotech, Birmingham, AL, USA) as the secondary antibody, and Lumitera (premixed chemiluminescence substrate; Cosmo Bio Co., Ltd., Tokyo, Japan) as chemiluminescence reagents were used.

### 4.7. LDL/HDL Assay

The serum-enriched fractions using CD9 antibody and IgG-immobilized magnetic beads were washed with PBS, the supernatant was discarded, and 200 µL of 1× assay diluent (from the HDL and LDL/VLDL cholesterol assay kit) was added to 40 µL each, and then 200 µL of LDL Precipitation Reagent (HDL and LDL/VLDL cholesterol assay lit) was added to each of them. They were mixed well, allowed to stand at room temperature for 10 min, and centrifuged at 2000× *g* for 20 min. The supernatant was used for the HDL assay, and the precipitate was used for the LDL assay. The supernatant was diluted with 1× assay diluent (50-fold dilution for serum and 25-fold dilution for magnetic beads) for measurement, and the precipitates were dissolved by adding 400 µL of PBS and then diluted with 1× assay diluent (50-fold dilution for serum and 25-fold dilution for magnetic beads) for measurement. For each 50 µL, 50 µL of the cholesterol reaction reagent (HDL and LDL/VLDL cholesterol assay kit) was added and incubated at 37 °C for 45 min before measurement at 530 nm for excitation and 590 nm for fluorescence.

## 5. Conclusions

In this study, antibodies against CD9 were covalently immobilized on magnetic beads, and the lipid components of human serum that interacted with CD9 antibodies were analyzed by LC/MS. Interestingly, MeOH extraction of serum recovered most of the phospholipids at the same level as IPA extraction, but almost no triglycerides were recovered. However, after enrichment with CD9 magnetic beads, triglycerides were measurable after MeOH extraction, as well as other phospholipids. The CD9 magnetic beads did not enrich LDL or HDL. At present, the implications of these findings remain, but it is possible that there are differences in the distribution and localization of triglycerides in serum and those enriched by CD9 magnetic beads.

## Figures and Tables

**Table 1:** Demographic characteristics of patients.

Disease	Group	Age	Female	Male
Alzheimer’s disease (Average age: 74.6)	Alzheimer’s 1	75.3	2	4
	Alzheimer’s 2	70.5	2	4
	Alzheimer’s 3	76.8	5	1
Major depression (Average age: 74.6)	Depression 1	68.8	2	4
	Depression 2	76.7	3	3
	Depression 3	78.3	5	1
Parkinson’s disease (Average age: 73.8)	Parkinson’s 1	74.3	4	2
	Parkinson’s 2	73.7	3	3
	Parkinson’s 3	73.3	2	4
Schizophrenia (Average age: 72.2)	Schizophrenia 1	72.5	3	3
	Schizophrenia 2	73.3	3	3
	Schizophrenia 3	70.7	2	4
Stroke (Average age: 74.7)	Stroke 1	75.5	4	2
	Stroke 2	72.2	1	5
	Stroke 3	76.5	3	3

Ages are represented by their mean values. The number of people for each sex are shown. None of the subjects overlapped between the groups.

## Data Availability

The data presented in this study are available on request from the corresponding author. The data are not publicly available due to raw LC-MS data files are too big to share easily to public, and we have security vulnerabilities.

## Supplementary Materials

The following supporting information can be downloaded at: www.mdpi.com/article/10.3390/metabo12030230/s1, Figure S1. Phospholipids and triglycerides profiles by disease in serum. The vertical axis shows the peak area for each lipid. Lipids are listed by class on the horizontal axis. Each peak area represents the average of three pooled serums (each pooled serum is a mixture of six sera), and the standard error of each peak is plotted. Dark blue, Alzheimer’s disease (AD); red, major depression (DP); gray, Parkinson’s disease (PD); yellow schizophrenia (SC); light blue, stroke (ST). (a) lipid profiles extracted directly from human serum. (b) Lipid profiles extracted after concentration of human serum with CD9 anti-body-immobilized magnetic beads. Figure S2. Triglycerides (TGs) profiles by disease in serum. The vertical axis shows the peak area for each TG. TGs are listed by class on the horizontal axis. Each peak area represents the average of three pooled serums (each pooled serum is a mixture of six sera), and the standard error of each peak is plotted. Dark blue, Alzheimer’s disease (AD); red, major depression (DP); gray, Parkinson’s disease (PD); yellow schizophrenia (SC); light blue, stroke (ST). (a) TG profiles extracted directly from human serum. (b) TG profiles extracted after concentration of human serum with CD9 anti-body-immobilized magnetic beads. Figure S3. Phosphatidylcholine (PC) profiles by disease in serum. The vertical axis shows the peak area for each PC. PCs are listed by class on the horizontal axis. Each peak area represents the average of three pooled serums (each pooled serum is a mixture of six sera), and the standard error of each peak is plotted. Dark blue, Alzheimer’s disease (AD); red, major depression (DP); gray, Parkinson’s disease (PD); yellow schizophrenia (SC); light blue, stroke (ST). (a) PC profiles extracted directly from human serum. (b) PC profiles extracted after concentration of human serum with CD9 anti-body-immobilized magnetic beads. Figure S4. Serum lysophosphatidylcholine (LPC) profile in serum. The vertical axis shows the peak area for each LPC. Alzheimer’s disease (AD); dark blue, major depression (DP); red, Parkinson’s disease (PD); gray, schizophrenia (SC); yellow, stroke (ST): light blue. LPC (X:Y) refers to the component containing X number of fat carbons and Y number of unsaturated bonds. All profiles are the averages of the three groups. Error bars mean S.E. (a) LPC extracted directly from human serum. (b) LPC extracted after concentration of human serum with CD9 anti-body-immobilized magnetic beads.
