# Supplementary material for: Lipid Profiles of Human Serum Fractions Enhanced with CD9 Antibody-Immobilized Magnetic Beads
Source: Metabolites. 2022 Mar 5;12(3):230. doi: 10.3390/metabo12030230 (PMC8956076; doi:10.3390/metabo12030230)
Supplement: Supplementary file 1 [file metabolites-12-00230-s001.zip › Supplementary Figure S3 PCprofile.pdf]

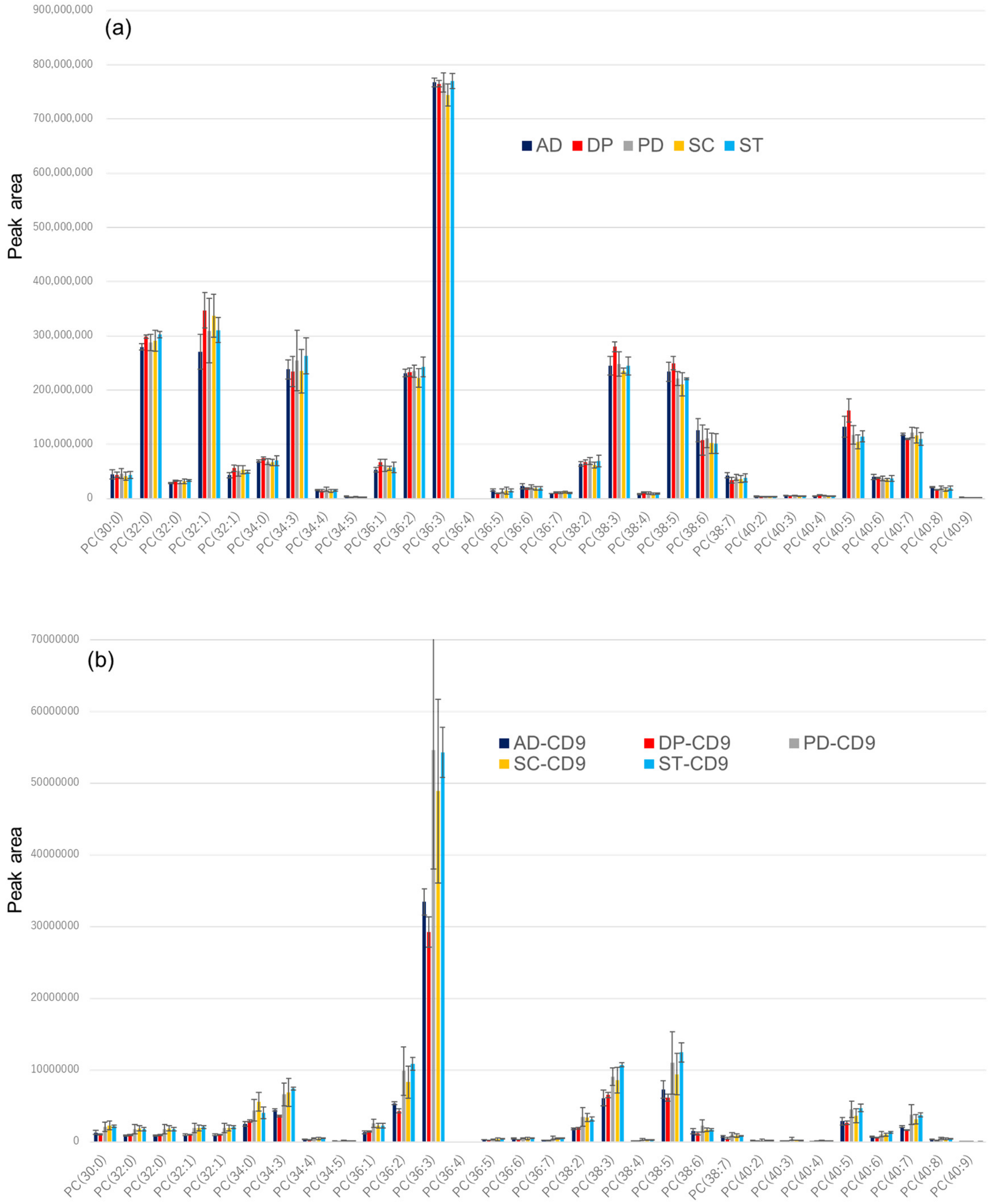

Supplementary Figure S3.

Phosphatidylcholine (PC) profiles by disease in serum. The vertical axis shows the peak area for each PC. PCs are listed by class on the horizontal axis. Each peak area represents the average of three pooled serums (each pooled serum is a mixture of six sera), and the standard error of each peak is plotted. Dark blue, Alzheimer's disease (AD); red, major depression (DP); gray, Parkinson's disease (PD); yellow schizophrenia (SC); light blue, stroke (ST). (a) PC profiles extracted directly from human serum. (b) PC profiles extracted after concentration of human serum with CD9 anti-body-immobilized magnetic beads.
