# Supplementary material for: Lipid Profiles of Human Serum Fractions Enhanced with CD9 Antibody-Immobilized Magnetic Beads
Source: Metabolites. 2022 Mar 5;12(3):230. doi: 10.3390/metabo12030230 (PMC8956076; doi:10.3390/metabo12030230)
Supplement: Supplementary file 1 [file metabolites-12-00230-s001.zip › Supplementary Figure S4 LPCs.pdf]

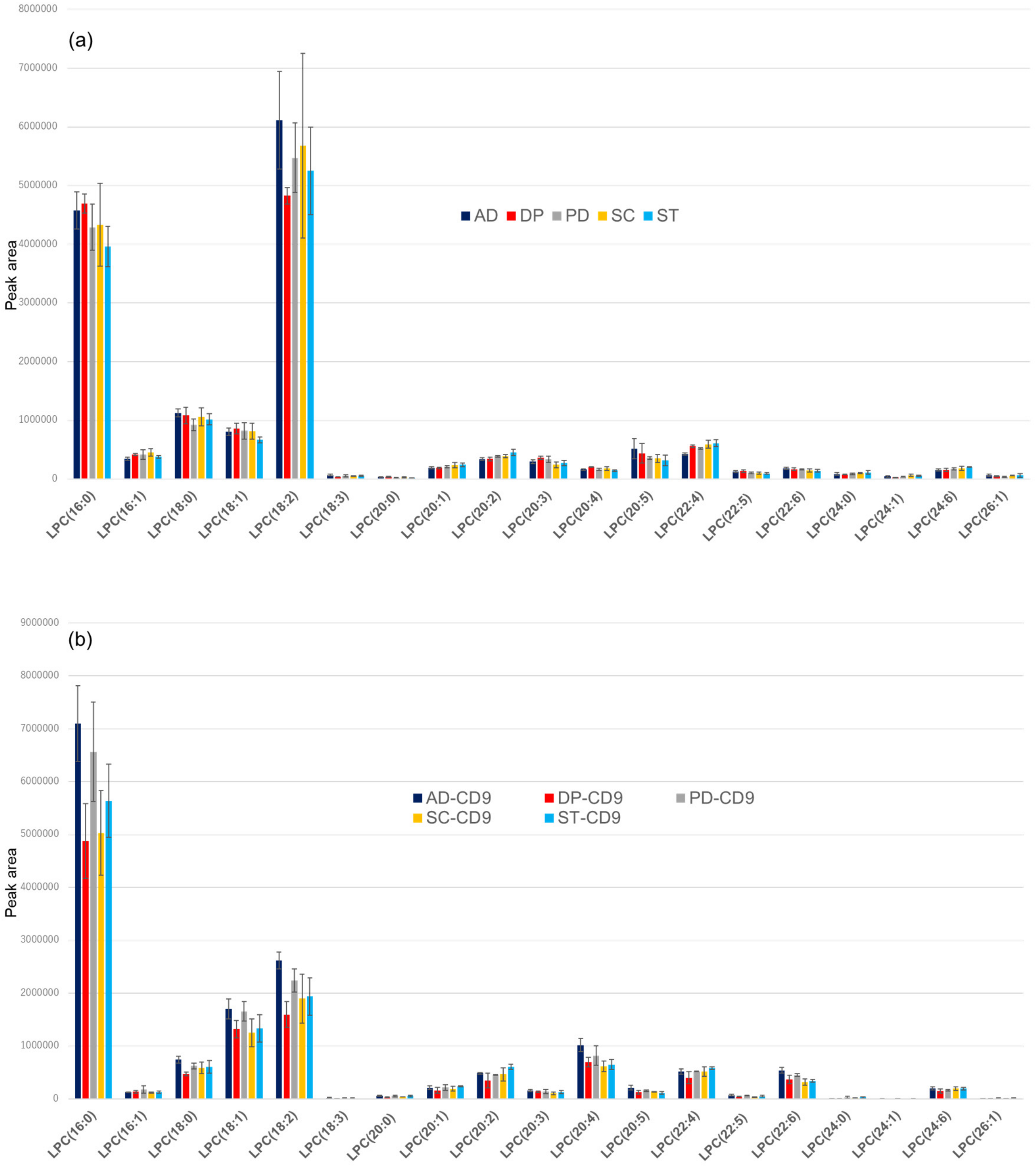

Supplementary Figure S4.

Serum lysophosphatidylcholine (LPC) profile in serum The vertical axis shows the peak area for each LPC. Alzheimer's disease (AD); dark blue, major depression (DP); red, Parkinson's disease (PD); gray, schizophrenia (SC); yellow, stroke (ST): light blue. LPC (X:Y) refers to the component containing X number of fat carbons and Y number of unsaturated bonds. All profiles are the averages of the three groups. Error bars mean S.E. (a) LPC extracted directly from human serum. (b) LPC extracted after concentration of human serum with CD9 antibody-immobilized magnetic beads.
